# Selection of Suitable Reference Genes for qPCR Gene Expression Analysis of HepG2 and L02 in Four Different Liver Cell Injured Models

**DOI:** 10.1155/2020/8926120

**Published:** 2020-07-14

**Authors:** Jiyu Chen, Zhenzhen Bao, Yanli Huang, Zhenglong Wang, Yucheng Zhao

**Affiliations:** ^1^School of pharmacy, Jiangsu Health Vocational College, Nanjing, Jiangsu, China; ^2^School of Life Science and Technology, China Pharmaceutical University, Nanjing, China; ^3^Jiangsu Key Laboratory of Bioactive Natural Product Research and State Key Laboratory of Natural Medicines, School of Traditional Chinese Pharmacy, China Pharmaceutical University, Nanjing, Jiangsu, China

## Abstract

Quantitative real-time PCR (qPCR) has become a widely used approach to analyze the expression level of selected genes. However, owing to variations in cell types and drug treatments, a suitable reference gene should be selected according to special experimental design. In this study, we investigated the expression level of ten candidate reference genes in hepatoma carcinoma cell (HepG2) and human hepatocyte cell line (L02) treated with ethanol (EtOH), hydrogen peroxide (H_2_O_2_), acetaminophen (APAP), and carbon tetrachloride (CCl_4_), respectively. To analyze raw cycle threshold values (Cp values) from qPCR run, three reference gene validation programs, including Bestkeeper, geNorm, and NormFinder, were used to evaluate the stability of ten candidate reference genes. The results showed that TATA-box binding protein (*TBP*) and tubulin beta 2a (*TUBB2a*) presented the highest stability for normalization under different treatments and were regarded as the most suitable reference genes of HepG2 and L02. In addition, this study not only identified the most stable reference genes of each treatment, but also suggested that *β*-actin (*ACTB*), glyceraldehade-3-phosphate dehydrogenase (*GAPDH*), tyrosine 3-monooxygenase/tryptophan 5-monooxygenase activation protein zeta (*YWHAZ*), and beta-2 microglobulin (*B2M*) were the least stable reference genes in HepG2 and L02. This work was the first report to systematically explore the stability of reference genes in injured models of HepG2 and L02.

## 1. Introduction

Quantitative real-time PCR (qPCR) is commonly used in analyzing gene expression levels owing to its credible precision and high-throughput competence [1, 2]. However, quantitative analysis of gene expressions is unavoidably affected by several factors such as sample amount, cell activity, RNA integrity, and cDNA quality [3–5]. Hence, in order to avoid quantitative errors and obtain a reliable experimental result, one or several reference genes should be applied as a suitable endogenous control for quantitative measurement of gene expression. Some literature indicated that at least three reference genes were needed to normalize the analysis of qPCR [6, 7]. In addition, numerous reports affirmed that the stability of reference genes might change based on various experimental designs and samples [8, 9]. Hence, a stable reference gene, which ensures the stability in various experimental conditions, should be identified.

Traditionally, *GAPDH* and *ACTB* are most frequently used for normalization; however, they have been demonstrated unsuitable for internal control because their stability varies in different experiments and samples [10–12]. For instance, in Li et al.'s [13] study, the mean Cp value of *GAPDH* was 23.88 in H2O2 treated human umbilical vein endothelial cells (HUVEC), while in cytokines treated HUVEC, the mean Cp values of *GAPDH* was distinctly below 20 [14]. Undoubtedly, the varied expressions of reference genes lead to the inaccuracy of results. Fortunately, an increasing number of researches have focused on selecting and identifying suitable reference genes of humans [15], plant [16], cell line [17], algae [18], animal [19], and bacteria [20]. However, a systematic research about the validation of suitable reference genes for liver cell (HepG2 and L02) injured models has not been reported.

HepG2 is an immortalized human hepatoma cell line, and L02 is an immortalized hepatocyte cell line [21, 22]. Additionally, HepG2 and L02 are widely accepted model systems for investigating hepatotoxicity, intracellular trafficking, and drug targeting in vitro [23–25]. Owing to the stability of reference genes varied with drug-treatments and differed in different cell lines [26]. Hence, in this study, we chose four liver cell injured models commonly used in pharmacology and toxicology: ethanol (EtOH) [27], hydrogen peroxide (H_2_O_2_) [28], acetaminophen (APAP) [29], and carbon tetrachloride (CCl_4_) [30], which represented alcoholic liver injury (EtOH), hepatic oxidative stress (H_2_O_2_), drug liver injury (APAP), and acute liver damage (CCl_4_), respectively, to find the most appropriate reference genes in different cell injured models of HepG2 and L02.

In this study, ten candidate reference genes, *ACTB, B2M, GAPDH, TUBB2a*, hypoxanthine phosphoribosyltransferase 1 (*HPRT1*), succinate dehydrogenase complex flavoprotein subunit A (*SDHA*), TBP, YWHAZ, cytochrome c isoform 1 (*CYC1*), and glucuronidase beta (*GUSB*), were selected to investigate the most stable reference genes for normalization in liver cell injured models. To evaluate the stability of candidate reference genes comprehensively, four types of experimental treatments (EtOH, H_2_O_2_, APAP, and CCl_4_) were investigated in two cell types (HepG2 and L02) *in vitro*. In addition, in order to analyze the correlation between different concentrations of drug treatment and expression levels of reference genes, we selected three groups of different concentrations (low dose group, middle dose group, and high dose group) for each treatment. All concentrations were chosen based on previous studies [31–36] which had performed a cell viability assay proving varying degrees cytotoxicity. To analyze the original data, three statistical algorithms named, geNorm [37], NormFinder [38], and Bestkeeper [39] were used based on the manufacturers' procedures. The calculation results of three kinds of software showed that *TBP* and *TUBB2a* were the most stable ones among all treatments. Moreover, geNorm was also used to calculate the optimal number of reference genes needed for normalization, and the results showed that it was sufficient for accuracy normalization to choose two reference genes in most groups. To our knowledge, this is the first study about the selection of the best reference genes in liver cell injured models, which would provide a proper choice of reference genes and guarantee a dependable result in liver cell injured model research.

## 2. Materials and Methods

### 2.1. Reagents

The ethanol (EtOH, 99.5% pure), hydrogen peroxide (H2O2, 30.0% pure), acetaminophen (APAP, 99.5% pure), and carbon tetrachloride (CCl_4_, 99.5% pure) were purchased from Aladdin Biochemical Technology Co., Ltd (Shanghai, China); Penicillin and streptomycin were obtained from Beyotime Institute of Biotechnology (Shanghai, China); Trypsin-EDTA Solution was purchased from Sangon Biotech (Shanghai, China).

### 2.2. Cell Culture and Treatment

The hepatoma carcinoma cells (HepG2) were obtained from the American Type Culture Collection (HB-8065), and the human hepatocyte cells were purchased from the Cell Bank of Type Culture Collection of the Chinese Academy of Sciences. Cells were grown in Dulbecco's Modified Eagle Medium (DMEM, Gibco), containing 100 U/ml penicillin-streptomycin and 10% fetal bovine serum (FBS, Bioind) under standard conditions (37°C and 5% CO_2_). The cells were grown to 80% confluence and then passaged using Trypsin-EDTA Solution (0.25% Trypsin, 0.02% EDTA). All cells were divided into four groups for treatments: (a) control group; (b) low dose group; (c) middle dose group; (d) high dose group. For HepG2, cells were treated with four different treatments, including ethanol (100 mM, 200 mM, 400 mM), H_2_O_2_ (200 *μ*M, 400 *μ*M, 800 *μ*M), APAP (2.5 mM, 5 mM, 10 mM), and CCl_4_ (0.1%, 0.2%, 0.4%). For L02, cells were treated with four different treatments, including ethanol (100 mM, 200 mM, 400 mM), H_2_O_2_ (100 *μ*M, 200 *μ*M, 400 *μ*M), APAP (2.5 mM, 5 mM, 10 mM), and CCl_4_ (0.05%, 0.1%, 0.2%). The CCl_4_ were dissolved into 0.25% DMSO and then were added to the serum-free DMEM; the ethanol, H_2_O_2_, and APAP were dissolved into serum-free DMEM directly. Cells were seeded in six-well plates before being subjected to treatments. For all groups, cells were incubated in the presence or absence of various treatments and different concentrations for 24 h.

### 2.3. Screening of Candidate Reference Genes and Primer Design

According to previous studies [9, 40], a total of ten candidate reference genes (*ACTB*, *B2M*, *GAPDH*, *TUBB2a*, *HPRT1*, *SDHA*, *TBP*, *YWHAZ*, *CYC1*, and *GUSB*) were selected to ascertain the best reference genes of HepG2 and L02 in liver cell injured conditions. The nucleotide sequences were downloaded, using Primer 5 to design primers. Full gene names and accession numbers, as well as primer length and intron-spanning primers, were listed in Table 1. The data of qPCR were repeated three times of biological and technical replicates.

### 2.4. Total RNA, DNA Extraction and cDNA Synthesis

Total RNA was extracted from HepG2 and L02 and purified using the RNAiso Plus total RNA kit (TransGen Biotech, Dalian, China) according to the manufacturer's instruction. And then, DNase I (Takara, Dalian, China) was added to the sample to eliminate DNA contamination for RNA purity. The purity of the total RNA was assessed by measuring the absorbance ration at 260/280 nm of the samples. In addition, the quality of the RNA was confirmed by agarose gel electrophoresis. Purified RNA was reverse transcribed immediately after extraction. For qPCR experiments, HiScript® Q RT SuperMix for qPCR Kit (Vazyme, Nanjing, China) and a quantity of 1 *μ*g total RNA were added into a 20 *μ*l reaction volume to synthesize cDNA.

### 2.5. Quantitative Real-Time PCR

The sample reaction was run in 96-well plate. Real-time quantitative PCR with AceQ qPCR SYBR Green Master Mix (Vazyme, Nangjing, China) was performed at LightCycler 480 (Roche Molecular Biochemicals, Mannheim, Germany). Each reaction system was 20 *μ*l, respectively. AceQ qPCR SYBR Green Master Mix 10 *μ*l, forward and reverse primers were 0.4 *μ*M each, template cDNA 2 *μ*l, and added ddH_2_O to the final volume of 20 *μ*l. Each sample was repeated 3 times. The optimizing reaction conditions of real-time quantitative PCR as follows: 1 cycle of 95°C for 5 min, 40 cycles of 95°C for 10 sec, and then 60°C for 30 sec.

### 2.6. Analysis of Reference Genes Stability

In order to evaluate the stability of ten selected reference genes, three reference gene validation programs (geNorm, NormFinder, and BestKeeper) were used under the manufacture's instruction. NormFinder was applied to calculate the stability value (*M*) for finding the steadiest candidate genes. For geNorm, the calculation could determine the optimal number of reference genes and, similar to geNorm, evaluate the stability of candidate genes. BestKeeper was based on the coefficient of variance (CV) and the standard deviation (SD) of the Cp values to assess the steadiness of reference genes. Three biological and technical repeats were used for different experimental conditions.

## 3. Results

### 3.1. Verification of the Primers Specificity

We used PCR to identify the specificity of the designed primers by agarose gel electrophoresis, as S2 and S3 Figs shows, the single band and peak of a melting curve indicated primers possessed the good specificity.

### 3.2. Evaluating Expression of the Reference Gene

**Figure 1:**
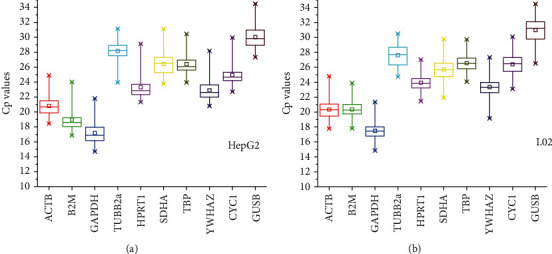
Expression levels of ten reference genes (*ACTB*, *B2M*, *GAPDH*, *TUBB2a*, *HPRT1*, *SDHA*, *TBP*, *YWHAZ*, *CYC1*, *GUSB*) in HepG2 (a) and L02 (b). Squares in the middle of the box represent the mean values, horizontal lines in the box represent the median, and whiskers represent the highest and lowest values.

The most suitable reference genes would have stable expression levels in various treatments and concentrations. And the Cp value of ten candidate reference genes underwent diver treatments were listed in Figure 1, ranging from 14.7 to 34.49 (HepG2 14.7 to 34.49, L02 14.85 to 34.48), suggesting that they have a noticeable variance in expression level. Particularly, most of the Cp values were in a range of 20 to 27. *ACTB*, *B2M*, *GAPDH*, *HPRT1*, and *YWHAZ* expressed lower Cp value around 20, while the rest of the genes showed that higher Cp value was greater than 25, especially the *GUSB*, which had the highest mean Cp values (HepG2 30.02 ± 1.49, L02 30.99 ± 1.66). Notably, *ACTB* showed the minimal change of Cp values from 18.44 to 24.90 under different treatments in HepG2, and meanwhile, the Cp values of *HPRT1* from 21.46 to 27.02 showed the low variation in L02, suggesting that the two genes might have a constant expression under various treatments and could be a suitable reference gene. In short, Cp values, combined with box-plot, presented the expression of the reference genes, and as well provided us a general understanding of gene stability.

### 3.3. Expression Stability of Candidate Reference Genes

The data obtained from different treatments (wild-type APAP, CCl_4_, ethanol, and H_2_O_2_) and each reference gene were analyzed with three Excel-based programs (geNorm, NormFinder, and BestKeeper) for further evaluation on the stability of putative reference genes.

### 3.4. geNorm Analysis

**Figure 2:**
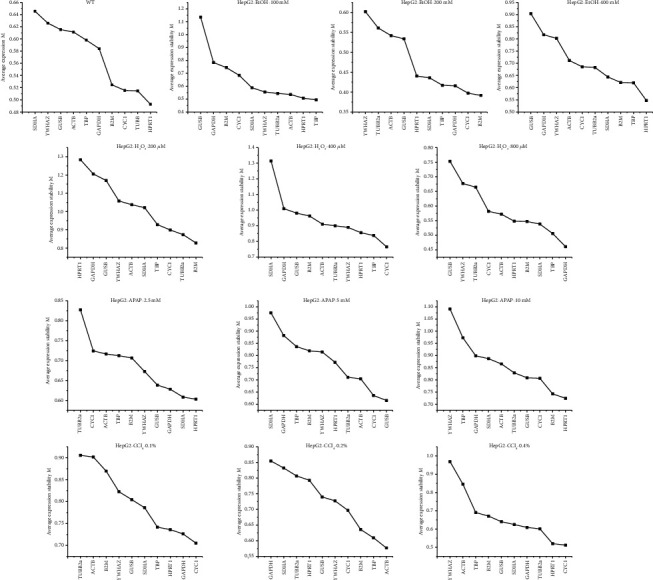
Expression stability of the reference genes in HepG2 evaluated by geNorm *M* values represents the average expression stability. From left to right, the value of *M* decreased in turn, indicating the stability gradually increased. Smaller *M* value means higher stability. The control group, ethanol, hydrogen peroxide, acetaminophen, and carbon tetrachloride were abbreviated to WT, EtOH, H_2_O_2_, APAP, and CCl_4_, respectively.

To ascertain the stability of candidate reference genes, geNorm was applied to evaluate the expression stability measurement (*M*) value by Cp values of each gene in groups. According to the analysis of geNorm, genes with the highest *M* values were considered as the least stable ones and the lowest the most. As shown in Figure 2 and S1 Figure, different reference genes had different *M* values in different treatments. For instance, in the L02 groups, *TBP* with the *M* value of 0.51 in 400 *μ*M H_2_O_2_ treatment would be the steadiest reference genes, while the *GUSB* was more than twice *TBP*, with *M* value was 1.37 in the same treatment. More interestingly, even the same reference gene had different expressions in different treatments. In the HepG2 groups, the gene with the lowest *M* values in 10 mM APAP treatment was *HPRT1*, which owned the highest *M* values in 200 *μ*M H_2_O_2_ treatment, meaning that *HPRT1* was the most stable genes in 10 mM APAP treatment and the least stable ones in 200 *μ*M H_2_O_2_ treatment.

### 3.5. NormFinder Analysis

NormFinder was used to evaluate the optimal gene for normalization in each experiment. The raw Cp values obtained from qPCR were firstly log-transformed and used as the input value for the NormFinder, and then used to analyze the expression stability according to the similarity of the expression profiles of candidate genes. Genes with lower values that were close to zero were regarded as the best candidate ones. As shown in Table 2 and S1 Table, the rank of *M* values was increasing from top to bottom of the table, whereas genes on the top of the table were the most stable reference genes. Therefore, the most stable candidate genes could be easily found from the table. In the HepG2 group, *CYC1* (7 times to be the top 3 candidate genes in 13 treatments) and *HPRT1* (8 times to be the top 3 in 13 treatments) were considered as the most stable reference genes; the results were similar to that of geNorm analysis. Nevertheless, in the L02 group, the results of geNorm and NormFinder analysis seemed to be different. For instance, in the NormFinder analysis, *TUBB2a* (9 times to be the top 3 in all treatments) was the steadiest ones, while *GAPDH*, the most stable genes in the geNorm analysis, appeared only once to be the top 3 of all treatments. Hence, the third analysis method should be used.

### 3.6. BestKeeper Analysis

BestKeeper was an Excel-based tool used to analyze the expression stability of the candidate reference gene. The standard deviation (SD) and coefficient of variation (CV) were calculated by BestKeeper to assess the stability of candidate reference genes in each group. Genes with the lowest SD and CV would be the most stable reference ones. As shown in Table 3 and S2 Table, the (CV ± SD) values of ten candidate reference genes progressively increased from top to bottom of tables, showing their decreasingly stability. As an example, TBP was listed on the top of Table 3, with a lower (CV ± SD) value of (0.85 ± 0.24), representing the most stable genes in 800 *μ*M H_2_O_2_ induced oxidative stress in HepG2, and meanwhile, *GUSB*, having a (CV ± SD) value of (5.42 ± 1.62), was listed at the bottom of the table. In HepG2 groups, some reference genes, namely, *TBP*, *CYC1*, and *TUBB2a*, might be the best suitable genes for the reason that they were listed on top 3 of the rank in majority treatments. Similarly, *CYC1* and *TBP* occupied most of the top 3 in the table, suggesting that the two candidate genes would be the steadiest genes in L02 treatments.

### 3.7. Optimal Numbers of Reference Genes for Normalization

**Figure 3:**
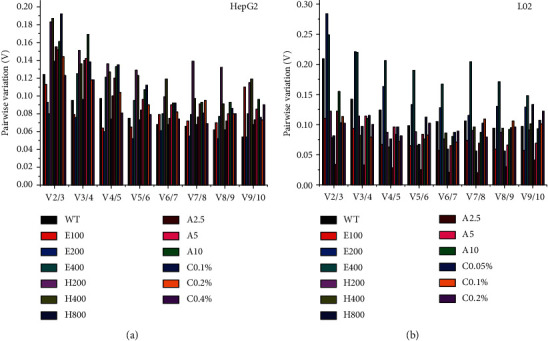
Calculation of the optimal number of reference genes for quantitative analysis using geNorm. Pairwise variation (Vn/*n* + 1) of reference genes analyzed in different treatments was listed in (a, b) set the cut-off threshold value 0.15, calculating the optimal number of reference genes for precise quantitative in this work. WT, E100 (E200, E400), H100 (H200, H400, H800), A2.5 (A5, A10), and C0.05% (C0.1%, C0.2%, C0.4%), respectively, were the abbreviation for the control group, EtOH-100 mM (200 mM, 400 mM), H_2_O_2_-100 *μ*M (200 *μ*M, 400 *μ*M, 800 *μ*M), APAP-2.5 mM (5 mM, 10 mM, 20 mM), CCl_4_-0.05% (0.1%, 0.2%, 0.4%).

The minimal numbers of reference genes for accurate normalization could also be determined by geNorm, according to the calculation of pairwise variation (variation coefficient, V) between the normalization factors (NF) in various treatment sets using Vn/*n* + 1 < 0.15 as a criterion cut-off value [37]. Based on this rule, the calculation was listed in Figure 3. As we can see, there were enough to choose two or three reference genes in most treatments of HepG2 and L02 for normalization. Moreover, 10 mM APAP treatment in HepG2, 200 mM EtOH, and 400 mM EtOH in L02, respectively, required four, five, and nine reference genes for normalization.

## 4. Discussion

Quantitative real-time PCR is one of the most accurate and commonly used techniques for analysis of gene transcript levels. Selection of suitable reference gene is indispensable to guarantee the accuracy and consistency of the data and minimize the experimental errors. To confirm the precise expression analysis of putative genes, numerous steady reference genes have been verified in different experimental designs [41, 42]. Hence, the purpose of this study was to investigate the expression stability of ten candidate reference genes (*ACTB*, *B2M*, *GAPDH*, *TUBB2a*, *HPRT1*, *SDHA*, *TBP*, *YWHAZ*, *CYC1*, *GUSB*) in two in-vitro cell types, namely, HepG2 cells and L02 cells, and determine the optimal candidate genes under the treatment of alcoholic liver injury (EtOH), hepatic oxidative stress (H_2_O_2_), drug liver injury (APAP), and acute liver damage (CCl_4_). After that, raw data was input and calculated in three Excel-based programs: geNorm, NormFinder, and BestKeeper.

The data from qPCR run of ten candidate genes were listed in Figure 1, where the expression level and mean Cp values of the candidate genes ranging from 14.7 to 34.49 (HepG2 14.7 to 34.49, L02 14.85 to 34.48) could be easily seen. However, the scope of the Cp values of some selected genes inconsistent with the previous study [43, 44] might be a result of liver damage treatments. Based on the fact that the genes with the highest expression levels owned minimal Cp values, *GAPDH* with the lowest mean Cp values of 17.47 in HepG2 and 17.25 in L02 means that the *GAPDH* was abundantly distributed in the two cell types. Considering that a wide distribution range tends to be low stability and moreover, Cp values with low variation would be more suitable for reference gene selection. The variation of Cp values suggested that *ACTB* and *HPRT1* were the best reference genes in HepG2 and L02, while *HPRT1* and *YWHAZ* were the least ones. The verification above was a little different from the calculation of the three Excel-based programs. For instance, the Cp values of *ACTB* and *HPRT1* might not fluctuate significantly, but in the calculations of the three kinds of software, the two genes appeared at the bottom of ranking frequently, which reflected more instability. Accordingly, more calculation results needed to be combined.

Furthermore, some literature reported that the expression level of reference genes would change under different concentration treatments [26, 45]. Hence, we investigated the stability of the reference genes in the same treatment of different concentrations with the results of three software calculations. Since the results of three software varied based on different algorithms, we selected the top five (six in some groups) reference genes for each calculation to evaluate them comprehensively. We identified the top five genes under three concentrations, and then found that genes were common in geNorm, NormFinder, and Bestkeeper. For instance, in CCl_4_ of three concentrations treated L02 cells, *GUSB*, *TUBB2a*, and *TBP* commonly appeared to be the top five of Bestkeeper calculation results, while *SDHA*, *GAPDH*, and *TUBB2a* of geNorm and *SDHA*, *TBP*, and *TUBB2a* of NormFinder, respectively. Apparently, *TUBB2a* appeared to be one of the top five of each calculation. Hence, we recommended *TUBB2a* as the most stable reference genes in CCl_4_ treated L02 cells. Likewise, in L02 cells, *TBP* was considered as the most stable genes in EtOH, H_2_O_2_, and APAP treatments, and *TUBB2a* was the steadiest in CCl_4_ treatments. In HepG2 cells, we suggested *TBP* being the most stable reference genes in EtOH and H_2_O_2_ treatments, and *GUSB* and *CYC1* in APAP and CCl_4_ treatments, respectively. In the same way, we evaluated the least stable reference genes in each group. On the whole, *ACTB*, *GAPDH*, *YWHAZ*, and *B2M* always ranked the last, meaning that they were considered as the least stable. Hence, we did not recommend *ACTB*, *GAPDH*, *YWHAZ*, or *B2M* as internal control for normalization. However, in Bridget's study [44], they identified *GAPDH* as the most stable reference genes in APAP treated HepG2, opposite to our research, which was mainly because they evaluated the stability only using geNorm, which might lead to inaccurate results.

Based on the analysis above, we found the best suitable reference genes in different treatments. Nevertheless, how many reference genes were required to be chosen for optimal data normalization required further investigation. Hence, we chose the geNorm software, which could calculate the optimal number of reference genes in a qPCR experiment to solve these problems. According to the handbook [37], the V score of 0.15 as a criterion value was recommended, and an additional gene was included until (Vn/Vn + 1) was lower than 0.15. In this study, the results showed that the majority of pairwise *V* values were lower than 0.15 after a total of 26 treated groups. and the calculation was shown in Figure 3, among which 23 out of 26 groups with a low *V* < 0.15, signifying the inclusion of additional reference genes was unnecessary in those 23 groups. Hence, two or three reference genes would suffice for reliable normalization in these groups above. Unfortunately, not all groups had a suitable *V* value of lower than 0.15. For instance, in the 400 mM EtOH treated L02 group, all of pairwise *V* values were greater than 0.15. Vandesompele et al. recommended that it was a waste of resources to quantify more genes than necessary, and hence, the *V* value (V6/7 and V9/10 values were close to 0.15) indicated that six reference genes should be a good choice for normalization in 400 mM treated L02 group.

## 5. Conclusion

In this study, 10 candidate genes were selected and evaluated in two types of liver cells (HepG2 and L02) for four types of liver cell injured treatments using the three different algorithms, namely, BestKeeper, geNorm, and NormFinder. To the best of our knowledge, this was the first systematic selection of reference genes in the liver cell injured model and laid the basis for further research in HepG2 and L02. Based on the analysis, we identified the best reference genes of HepG2 and L02 under the treatments of EtOH, H_2_O_2_, APAP, and CCl_4_. The results of gene expression revealed that *TBP* and *TUBB2a* were the most stable reference genes for normalization in different treatments. On one hand, in the HepG2, the most stable reference genes of EtOH and H_2_O_2_ treatments were *TBP*, while *GUSB* and *CYC1* were, respectively, the most suitable reference genes of APAP and CCl_4_ treatments. In the L02, *TBP* was identified as the most stable reference genes of EtOH, H_2_O_2_, and APAP treatments, while *TUBB2a* was the steadiest reference genes of CCl_4_ treatment. On the other hand, *ACTB*, *GAPDH*, *YWHAZ*, and *B2M* were the least stable reference genes in EtOH, H_2_O_2_, APAP, and CCl_4_ treated HepG2 and L02. In short, our study provided a credible selection of reference gene in HepG2 and L02 injured models.

## Figures and Tables

**Table 1:** Details of the ten candidate reference genes and primers used in the qPCR.

Gene	Description	Primer: forward/reverse(5′-3′)	Length (bp)	Accession number
ACTB	*β*-Actin	F: AAGGCCAACCGCGAGAAGAT R: GCCAGAGGCGTACAGGGATA	102	NM_001101
B2M	Beta-2 microglobulin	F: GTTTACTCACGTCATCCAGC R:AGACAAGTCTGAATGCTCCA	141	NM_004048
GAPDH	Glyceraldehade-3-phosphate dehydrogenase	F: GCCTCCTGCACCACCAACTG R: CCATCACGCCACAGTTTCCC	149	NM_002046
TUBB2a	Tubulin beta 2a	F: AACGCCACCCTCTCTGTCCA R: GCCGACACCAGGTGGTTGAG	143	NM_001069
HPRT1	Hypoxanthine phosphoribosyltransferase 1	F: ACTGAACGTCTTGCTCGAGA R: TGATGTAATCCAGCAGGTCA	112	NM_000194
SDHA	Succinate dehydrogenase complex flavoprotein subunit A	F: AAAGATCACGTCTACCTGCA R: CATGTTATAATGCACGGTGG	150	NM_004168
TBP	TATA-box binding protein	F: GTTCAGCAGTCAACGTCCCA R: TCATGGGGGAGGGATACAGT	127	NM_003194
YWHAZ	Tyrosine 3-monooxygenase/tryptophan 5-monooxygenase activation protein zeta	F: CAGGCTGAGCGATATGATGA R: CCTACGGGCTCCTACAACAT	126	NM_003406
CYC1	Cytochrome c isoform 1	F: CCAAAACCATACCCCAACAG R: AGTCCTCACCACCATGCCTA	103	NM_001916
GUSB	Glucuronidase beta	F: GTTCCTTTTGCGAGAGAGAT R: ACACGCAGGTGGTATCAGTC	124	NM_000181

**Table 2:** Expression stability values of ten candidate reference genes in HepG2 analyzed by NormFinder.

Rank	WT	EtOH 100 mM	EtOH 200 mM	EtOH 400 mM	H_2_O_2_ 200 *μ*M	H_2_O_2_ 400 *μ*M	H_2_O_2_ 800 *μ*M	APAP 2.5 mM	APAP 5 mM	APAP 10 mM	CCl_4_ 0.1%	CCl_4_ 0.2%	CCl_4_ 0.4%
1	HPRT1 0.011	HPRT1 0.004	CYC1 0.007	HPRT1 0.008	B2M 0.018	CYC1 0.013	TBP 0.011	HPRT1 0.013	GUSB 0.004	HPRT1 0.012	TBP 0.015	TBP 0.011	HPRT1 0.005
2	TUBB2a 0.012	TBP 0.005	TBP 0.009	TUBB2a 0.013	TUBB2a 0.019	TUBB2a 0.017	GAPDH 0.011	GUSB 0.015	CYC1 0.010	B2M 0.018	CYC1 0.017	ACTB 0.013	CYC1 0.008
3	CYC1 0.013	TUBB2a 0.005	SDHA 0.011	B2M 0.014	CYC1 0.020	TBP 0.019	SDHA 0.012	SDHA 0.018	TUBB2a 0.014	SDHA 0.019	SDHA 0.020	B2M 0.021	GUSB 0.009
4	B2M 0.017	YWHAZ 0.012	B2M 0.011	TBP 0.016	TBP 0.027	HPRT1 0.024	HPRT1 0.015	YWHAZ 0.022	ACTB 0.020	TUBB2a 0.020	HPRT1 0.021	TUBB2a 0.021	TUBB2a 0.016
5	TBP 0.018	SDHA 0.013	HPRT1 0.013	SDHA 0.018	GUSB 0.030	GUSB 0.025	B2M 0.017	CYC1 0.023	HPRT1 0.021	CYC1 0.022	TUBB2a 0.024	CYC1 0.021	SDHA 0.019
6	SDHA 0.019	ACTB 0.014	GAPDH 0.015	CYC1 0.020	SDHA 0.030	YWHAZ 0.027	TUBB2a 0.017	TBP 0.024	TBP 0.022	ACTB 0.024	GUSB 0.024	GUSB 0.022	TBP 0.024
7	ACTB 0.020	CYC1 0.022	GUSB 0.015	ACTB 0.025	YWHAZ 0.037	ACTB 0.031	CYC1 0.017	ACTB 0.028	YWHAZ 0.025	GUSB 0.025	YWHAZ 0.028	YWHAZ 0.022	B2M 0.029
8	GUSB 0.021	B2M 0.032	TUBB2a 0.016	GUSB 0.027	ACTB 0.039	B2M 0.037	ACTB 0.019	GAPDH 0.029	B2M 0.029	TBP 0.029	GAPDH 0.032	SDHA 0.024	GAPDH 0.031
9	YWHAZ 0.023	GAPDH 0.041	ACTB 0.020	YWHAZ 0.028	HPRT1 0.046	SDHA 0.043	GUSB 0.022	TUBB2a 0.030	SDHA 0.031	GAPDH 0.035	B2M 0.034	HPRT1 0.029	ACTB 0.037
10	GAPDH 0.029	GUSB 0.042	YWHAZ 0.023	GAPDH 0.041	GAPDH 0.061	GAPDH 0.048	YWHAZ 0.024	B2M 0.032	GAPDH 0.039	YWHAZ 0.037	ACTB 0.037	GAPDH 0.044	YWHAZ 0.039

**Table 3:** Expression stability values of the reference genes calculated by BestKeeper in HepG2.

Rank	WT	EtOH 100 mM	EtOH 200 mM	EtOH 400 mM	H_2_O_2_ 200 *μ*M	H_2_O_2_ 400 *μ*M	H_2_O_2_ 800 *μ*M	APAP 2.5 mM	APAP 5 mM	APAP 10 mM	CCl_4_ 0.1%	CCl_4_ 0.2%	CCl_4_ 0.4%
1	GUSB 1.57 ± 0.45	HPRT1 1.10 ± 0.24	SDHA 0.74 ± 0.20	B2M 1.57 ± 0.29	CYC1 1.37 ± 0.33	CYC1 1.49 ± 0.36	TBP 0.85 ± 0.24	SDHA 1.78 ± 0.44	CYC1 0.95 ± 0.25	GUSB 1.75 ± 0.59	GUSB 0.90 ± 0.26	HPRT1 1.67 ± 0.38	TBP 1.15 ± 0.30
2	ACTB 1.89 ± 0.39	CYC1 1.24 ± 0.3	TBP 1.00 ± 0.26	TBP 1.67 ± 0.44	TUBB2a 1.68 ± 0.48	YWHAZ 1.51 ± 0.35	CYC1 0.95 ± 0.24	TBP 2.50 ± 0.63	GUSB 1.47 ± 0.46	CYC1 2.60 ± 0.75	CYC1 1.10 ± 0.27	GUSB 1.68 ± 0.51	SDHA 1.53 ± 0.38
3	TUBB2a 2.22 ± 0.63	TBP 1.28 ± 0.33	CYC1 1.08 ± 0.27	SDHA 1.70 ± 0.46	SDHA 1.90 ± 0.51	GUSB 1.54 ± 0.47	B2M 1.08 ± 0.21	TUBB2a 2.83 ± 0.72	TUBB2a 1.59 ± 0.45	TUBB2a 3.25 ± 0.94	HPRT1 1.43 ± 0.32	TBP 1.72 ± 0.45	CYC1 1.72 ± 0.43
4	CYC1 2.41 ± 0.57	SDHA 1.79 ± 0.47	GUSB 1.12 ± 0.34	YWHAZ 2.08 ± 0.47	TBP 2.28 ± 0.61	HPRT1 1.77 ± 0.40	SDHA 1.16 ± 0.32	GUSB 2.97 ± 0.86	HPRT1 1.64 ± 0.41	ACTB 3.27 ± 0.77	B2M 1.99 ± 0.37	CYC1 1.84 ± 0.47	TUBB2a 1.97 ± 0.54
5	B2M 2.65 ± 0.48	TUBB2a 1.92 ± 0.54	ACTB 1.16 ± 0.25	HPRT1 2.12 ± 0.49	ACTB 2.58 ± 0.53	TUBB2a 1.80 ± 0.52	HPRT1 1.22 ± 0.29	YWHAZ 3.25 ± 0.71	B2M 1.74 ± 0.35	TBP 3.38 ± 0.98	TBP 2.19 ± 0.57	YWHAZ 2.40 ± 0.54	HPRT1 2.31 ± 0.53
6	TBP 2.78 ± 0.72	ACTB 2.01 ± 0.41	B2M 1.19 ± 0.22	CYC1 2.16 ± 0.53	B2M 2.84 ± 0.51	TBP 1.84 ± 0.49	GAPDH 1.39 ± 0.25	HPRT1 3.28 ± 0.76	YWHAZ 2.17 ± 0.53	B2M 3.90 ± 0.88	SDHA 2.42 ± 0.61	TUBB2a 2.58 ± 0.73	GUSB 2.72 ± 0.81
7	HPRT1 3.10 ± 0.70	YWHAZ 2.13 ± 0.47	TUBB2a 1.28 ± 0.37	TUBB2a 2.20 ± 0.62	GUSB 2.96 ± 0.88	ACTB 2.78 ± 0.57	GUSB 1.59 ± 0.50	CYC1 3.31 ± 0.82	ACTB 2.20 ± 0.50	HPRT1 4.04 ± 1.11	YWHAZ 2.45 ± 0.54	ACTB 2.84 ± 0.57	YWHAZ 2.97 ± 0.67
8	SDHA 3.41 ± 0.91	GUSB 2.18 ± 0.65	GAPDH 1.45 ± 0.25	GUSB 2.58 ± 0.78	YWHAZ 3.48 ± 0.78	B2M 3.14 ± 0.59	TUBB2a 1.62 ± 0.47	ACTB 3.39 ± 0.67	TBP 2.59 ± 0.72	SDHA 4.41 ± 1.30	TUBB2a 2.63 ± 0.74	B2M 2.92 ± 0.55	ACTB 3.92 ± 0.75
9	YWHAZ 3.63 ± 0.82	B2M 2.82 ± 0.51	HPRT1 1.55 ± 0.35	ACTB 3.37 ± 0.71	HPRT1 4.86 ± 1.10	SDHA 3.84 ± 1.05	ACTB 1.65 ± 0.36	B2M 4.86 ± 0.92	SDHA 2.68 ± 0.73	GAPDH 4.81 ± 0.97	ACTB 2.95 ± 0.60	SDHA 3.32 ± 0.84	B2M 4.41 ± 0.83
10	GAPDH 3.99 ± 0.66	GAPDH 3.30 ± 0.55	YWHAZ 2.32 ± 0.54	GAPDH 4.03 ± 0.71	GAPDH 5.26 ± 0.89	GAPDH 4.21 ± 0.71	YWHAZ 2.24 ± 0.53	GAPDH 4.95 ± 0.78	GAPDH 3.43 ± 0.63	YWHAZ 6.38 ± 1.64	GAPDH 3.42 ± 0.57	GAPDH 4.38 ± 0.73	GAPDH 4.46 ± 0.73

## Data Availability

The data used to support the findings of this study are included within the article.

## Supplementary Materials

Supplementary Materials S1 Figure: expression stability of the housekeeping genes in L02 evaluated by geNorm *M* values represents the average expression stability. From left to right, the value of *M* decreased in turn, indicating the stability gradually increased. Smaller *M* value means higher stability. The control group, ethanol, hydrogen peroxide, acetaminophen, and carbon tetrachloride were abbreviated to WT, EtOH, H2O2, APAP, and CCl4, respectively. S2 Figure: agarose gel (1%) electrophoresis of the ten candidate housekeeping genes. 1-10 represent ACTB, B2M, GAPDH, TUBB2a, HPRT1, SDHA, TBP, YWHAZ, CYC1, and GUSB, respectively. S3 Figure: melt curves of the ten candidate housekeeping genes. S1 Table: expression stability values of ten candidate housekeeping genes in L02 analyzed by NormFinder. S2 Table: expression stability values of the housekeeping genes calculated by BestKeeper in L02.
