# Supplementary material for: Selection of Suitable Reference Genes for qPCR Gene Expression Analysis of HepG2 and L02 in Four Different Liver Cell Injured Models
Source: Biomed Res Int. 2020 Jul 14;2020:8926120. doi: 10.1155/2020/8926120 (PMC7376413; doi:10.1155/2020/8926120)

S1 Figure Expression stability of the housekeeping genes in L02 evaluated by geNorm M values represent the average expression stability. From left to right, the value of M decreased in turn, indicating the stability gradually increased. Smaller M value means higher stability. The control group, ethanol, hydrogen peroxide, acetaminophen and carbon tetrachloride were abbreviated to WT, EtOH, H2O2, APAP and CCl4 respectively.

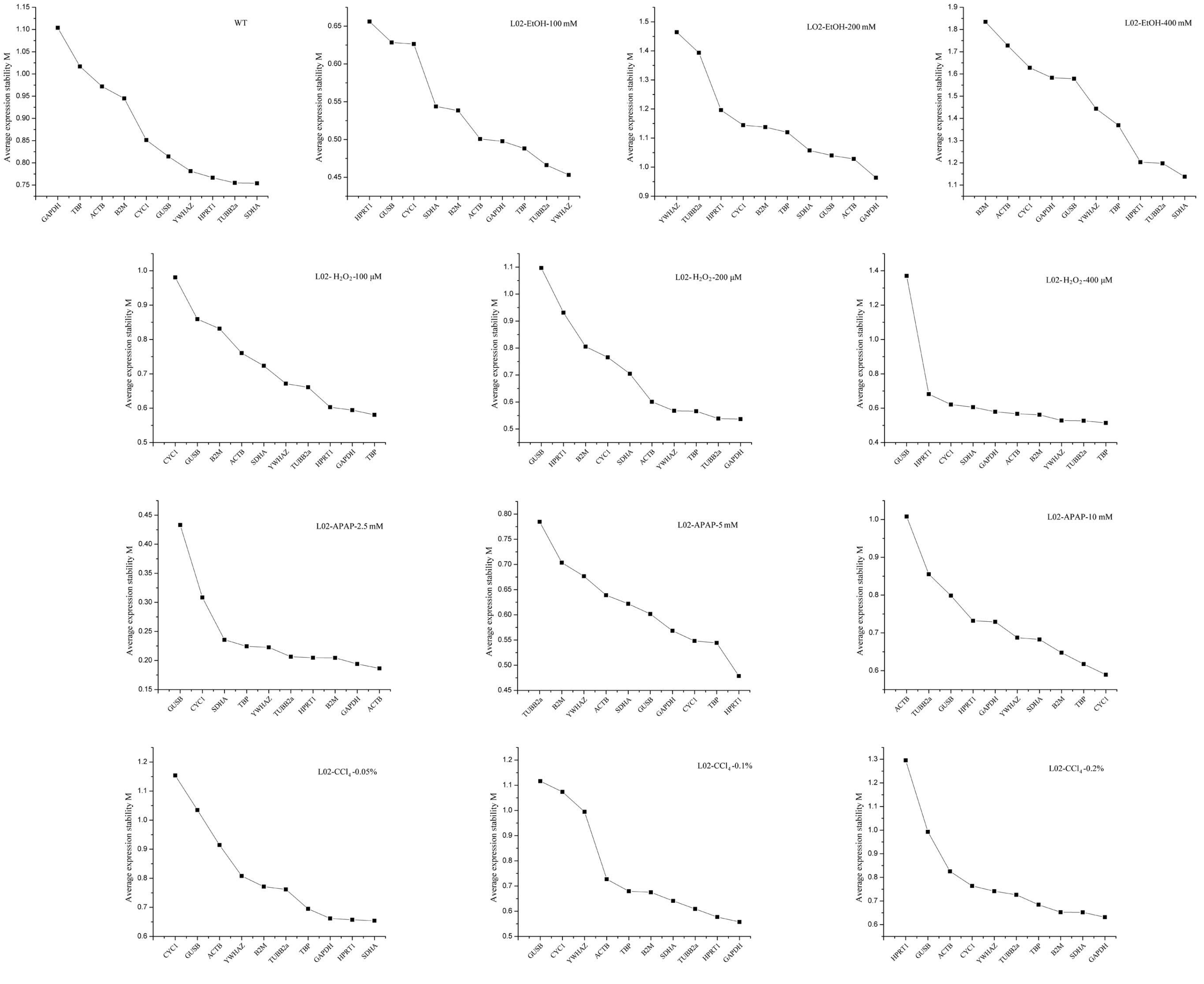

S2 Figure Agarose gel (1%) electrophresis of the ten candidate housekeeping genes. 1-10 represent ACTB, B2M, GAPDH, TUBB2a, HPRT1, SDHA, TBP, YWHAZ, CYC1 and GUSB, respectively

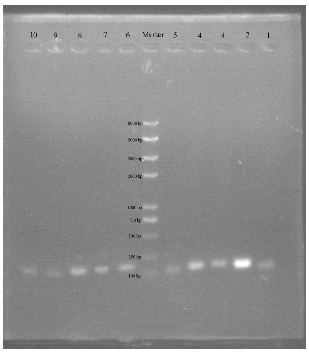

S3 Figure Melt curves of the ten candidate housekeeping genes.

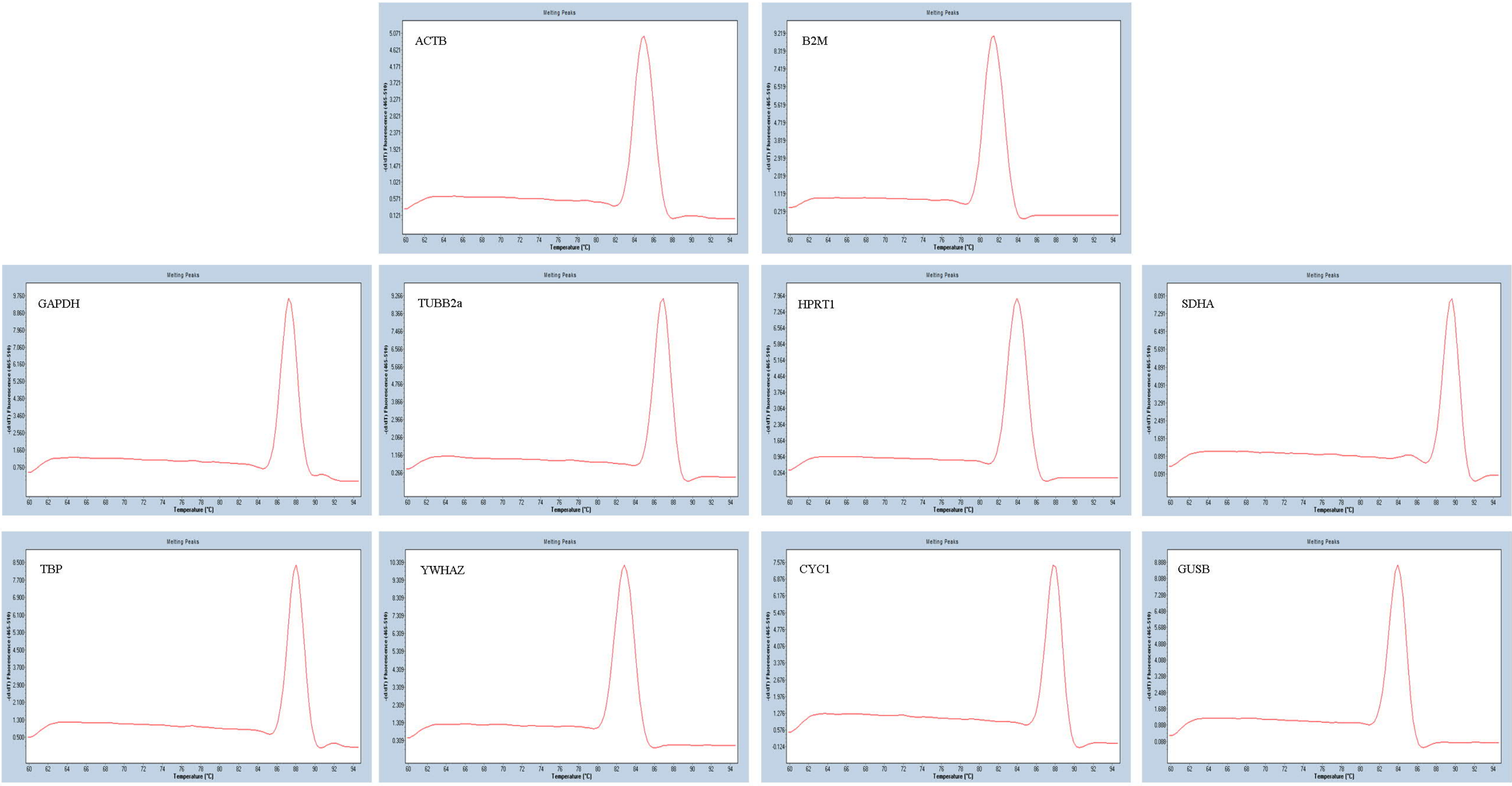

Supplement: Supplementary Materials — S1 Figure: expression stability of the housekeeping genes in L02 evaluated by geNorm M values represents the average expression stability. From left to right, the value of M decreased in turn, indicating the stability gradually increased. Smaller M value means higher stability. The control group, ethanol, hydrogen peroxide, acetaminophen, and carbon tetrachloride were abbreviated to WT, EtOH, H2O2, APAP, and CCl4, respectively. S2 Figure: agarose gel (1%) electrophoresis of the ten candidate housekeeping genes. 1-10 represent ACTB, B2M, GAPDH, TUBB2a, HPRT1, SDHA, TBP, YWHAZ, CYC1, and GUSB, respectively. S3 Figure: melt curves of the ten candidate housekeeping genes. S1 Table: expression stability values of ten candidate housekeeping genes in L02 analyzed by NormFinder. S2 Table: expression stability values of the housekeeping genes calculated by BestKeeper in L02. [file 8926120.f1.pdf]
